# Increasing the stability of DNA nanostructure templates by atomic layer deposition of Al_2_O_3_ and its application in imprinting lithography

**DOI:** 10.3762/bjnano.8.236

**Published:** 2017-11-09

**Authors:** Hyojeong Kim, Kristin Arbutina, Anqin Xu, Haitao Liu

**Affiliations:** 1 Department of Chemistry, University of Pittsburgh, 219 Parkman Avenue, Pittsburgh, Pennsylvania 15260, United States of America

**Keywords:** aluminium oxide (Al_2_O_3_), atomic layer deposition, DNA nanostructure, nanofabrication, nanoimprint lithography, pattern transfer, polymer stamp, replica molding

## Abstract

We present a method to increase the stability of DNA nanostructure templates through conformal coating with a nanometer-thin protective inorganic oxide layer created using atomic layer deposition (ALD). DNA nanotubes and origami triangles were coated with ca. 2 nm to ca. 20 nm of Al_2_O_3_. Nanoscale features of the DNA nanostructures were preserved after the ALD coating and the patterns are resistive to UV/O_3_ oxidation. The ALD-coated DNA templates were used for a direct pattern transfer to poly(L-lactic acid) films.

## Introduction

In 1982, Seeman et al. first introduced the idea of utilizing DNA to build a mechanically robust nanostructure [1]. Since then, the field of structural DNA nanotechnology has evolved remarkably from immobile Holliday junctions to complex shapes fabricated from single-stranded tiles [2–3]. Through rational design, the self-assembly of DNA can be brought into almost any shape with nanometer-scale precision and accuracy. Examples of such structures are one-dimensional (1D) [4–7], two-dimensional (2D) [8–11] and three-dimensional (3D) [12–15] nanostructures with diverse and complex features. Therefore, self-assembled DNA nanostructures are considered to be an ideal template for nanofabrication because it is easy to control their structural complexity and diversity at the nanoscale.

Many approaches have been developed to use DNA nanostructures as templates to pattern a wide range of materials, such as proteins [16–19], carbon nanotubes [20–23] and metal nanoparticles through the direct assembly of these materials onto the DNA nanostructures [16,18,24–29]. The metallized DNA nanostructures have been used to pattern graphene [30]. DNA nanostructures have also been used as masks. The patterns of 1D DNA nanotubes and 2D DNA arrays were replicated to metal films by metal evaporation onto the DNA nanostructures and subsequent lift-off of the metal films [31]. Aligned DNA molecular bundles became shadow masks for angled metal vapor deposition and the exposed substrate through shadow gaps was etched to generate trenches with linewidths of sub-10 nm resolution [32]. By differentiating the adsorption of water between DNA nanostructures and a SiO_2_ substrate, the rates of HF vapor-phase etching of the SiO_2_ substrate [33] and of chemical vapor deposition of SiO_2_ and TiO_2_ on the DNA nanostructures and the substrate [34] were modulated to replicate the patterns of the DNA nanostructures into those of the inorganic oxides. In both cases, the patterns of the nanostructures were transferred in both positive tone and negative tone at room temperature. Similarly, DNA nanostructures were also used in the anhydrous HF vapor etching of a SiO_2_ substrate, producing positive imprints of the DNA nanostructures with sub-10 nm resolution [35]. DNA nanostructures were also converted into carbon nanostructures with shape conservation by atomic layer deposition of Al_2_O_3_ onto the nanostructures followed by thermal annealing [36]. In addition to the 2D pattern transfer processes, gold nanoparticles with specified 3D shapes were synthesized by growing seed particles in the internal cavities of 3D DNA nanostructures [37–38].

Compared to the above developments, there are only a limited number of studies of the use of DNA nanostructures as master templates for soft lithography. Soft lithography relies on elastomeric stamps or molds bearing fine features of relief on their surfaces to transfer patterns [39]. The spatial resolution and diverse features of the relief structures on the stamps intrinsically limit the application of soft lithography. Thus, the preparation of master templates, where the stamps are derived, has become an important research area. State-of-the-art technologies for fabrication of the master templates are deep ultraviolet lithography (DUL) and electron-beam (e-beam) lithography. However, both of these lithography techniques are not suitable to provide sub-10 nm resolution. DUL with ArF lasers (λ = 193 nm) and water immersion lenses is not able to provide a structure with spacing less than 40 nm because of its diffraction-limited resolution [40]. Although e-beam lithography is capable of reaching resolutions below 10 nm [41], it is difficult to produce the master templates in larger numbers because of its high cost [42–44]. In 2015, the aligned patterns of natural salmon milt DNA bundles were first transferred to negative replicas on unsaturated polyester resins, which were further used to pattern positive replicas on water-swollen polyacrylamide gels [45]. However, the shape of the DNA bundles is limited to 1D patterns, and their dimensions are relatively large compared to the resolution of the state-of-the-art lithographic techniques. The average height and width of the DNA bundles were 90.53 ± 3.08 nm and 878.84 ± 22.79 nm, respectively.

Taking one step further in this direction, we have recently used DNA nanostructures as master templates for a direct pattern transfer to polymers with high diversity, complexity, and fidelity [46]. A wide range of DNA nanostructures, including DNA nanotubes, 1D λ-DNA, 2D DNA brick crystals with 3D features, hexagonal DNA 2D arrays, and DNA origami triangles, were tested for the pattern replication process to poly(methyl methacrylate) (PMMA), poly(L-lactic acid) (PLLA), and photo-cross-linked acryloxy perfluoropolyether (a-PFPE). The resulting negative imprints of the DNA nanostructures on the PMMA and PLLA polymer stamps further served as molds to transfer the patterns to positive imprints on a-PFPE films. In our method, the separation of the polymer film from the DNA nanostructure master template relies on using water to lower the adhesion between the film and the template. The key advantage of our method is that any polymer with hydrophobicity and/or low surface energy can be patterned with the DNA nanostructure master template. Furthermore, because the method uses spin-coating instead of hot-pressing, it is compatible with polymers having a wide range of glass transition temperatures (*T*_g_).

With our method, polymer stamps can be made with nanoscale features of dimensions ranging from several tens of nanometers to micrometers by logically designing and synthesizing DNA nanostructures. Our approach has one substantial technical problem, however, which is that the DNA nanostructure master templates cannot be used in a repetitive manner. The DNA nanostructures were partially damaged during the release of the PMMA and PLLA hydrophobic stamps from the hydrophilic master template. It still remains a challenge to develop an approach to increase the stability of the DNA nanostructure master templates.

In this paper, we establish a method to increase the chemical and/or mechanical stability of DNA nanostructure master templates by a nanometer-thin conformal coating of a protective inorganic oxide film grown by atomic layer deposition (ALD). We test the stability of DNA nanotube master templates with an Al_2_O_3_ layer against repeated pattern transfer, long-term storage and exposure to UV/O_3_. The effect of the thickness of the Al_2_O_3_ layer on the qualities of pattern transfer and shape conservation is also explored.

## Result and Discussion

**Figure 1:**
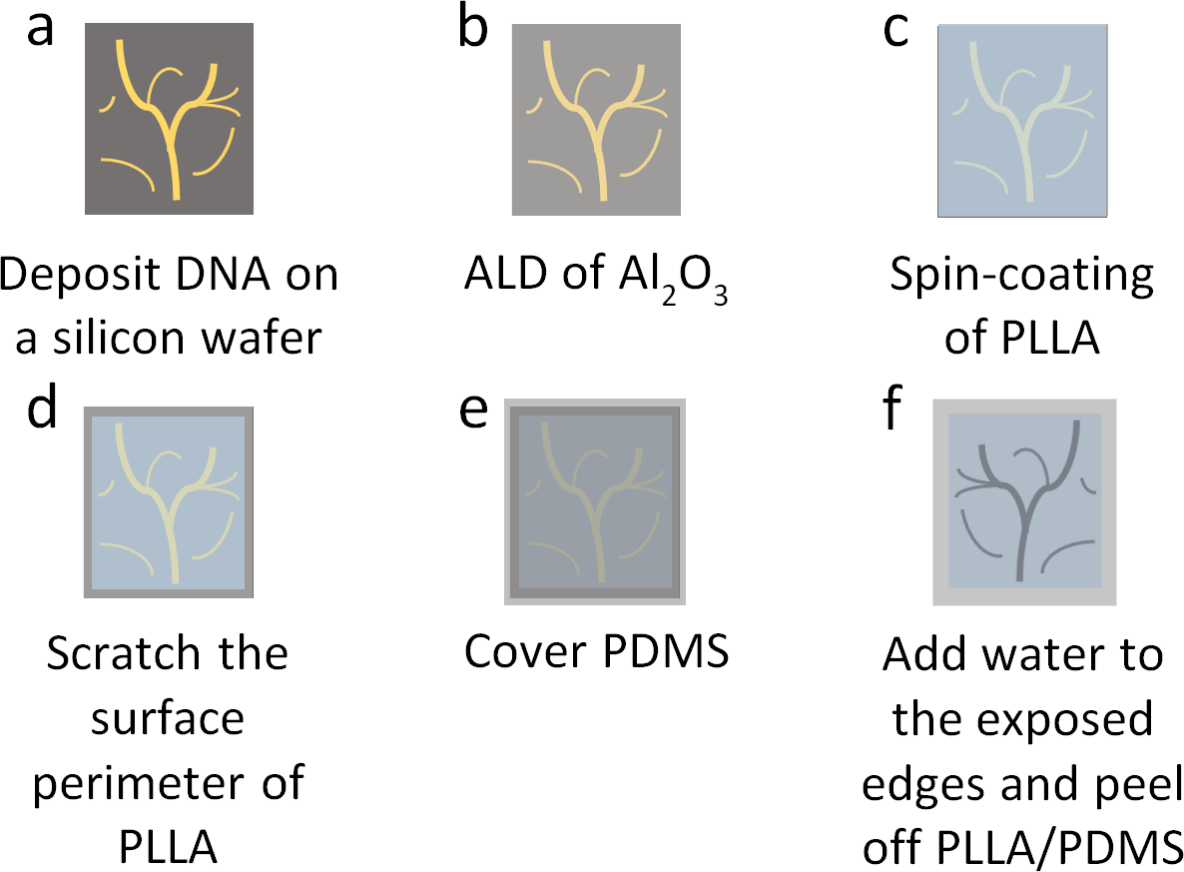
Fabrication process of a polymer stamp using a DNA nanostructure master template with a protective Al_2_O_3_ film. (a) DNA nanostructures are deposited on a silicon wafer. (b) The silicon wafer is coated with Al_2_O_3_ by atomic layer deposition. (c) A polymer film (e.g., PLLA) is spin-coated onto the silicon wafer. (d) The edges of the polymer film is scraped off with a blade. (e) A PDMS film is adhered to the polymer film as a backing support. (f) Droplets of water are added to the exposed edges of the silicon wafer and the PLLA/PDMS film is peeled off.

A DNA nanostructure master template with a protective Al_2_O_3_ film and a corresponding PLLA stamp were adapted from our previously published method [46] and the fabrication process is shown in Figure 1. DNA nanostructures were deposited onto a silicon wafer that was cleaned by piranha solution (Figure 1a). The entire surface of the DNA nanostructure master template was coated with a layer of Al_2_O_3_ by ALD (Figure 1b). After the ALD process, PLLA solution in dichloromethane (3 wt %) was spin-coated onto the template to prepare a PLLA film (Figure 1c). Around the edges of the silicon wafer, the PLLA film was scraped off with a blade and the silicon wafer underneath the PLLA film was revealed (Figure 1d). A polydimethylsiloxane (PDMS) film was placed on top of the PLLA film serving as a flexible backing to assist in the separation of the polymer film from the template (Figure 1e). Droplets of water were added to the exposed edges of the template, separating the hydrophobic PLLA film from the hydrophilic master template by penetration into the interface between them. After one minute, the PLLA/PDMS film was peeled off and the negative replica of the positive pattern of the DNA nanostructure master template formed on the surface of the film that was in contact with the DNA (Figure 1f).

**Figure 2:**
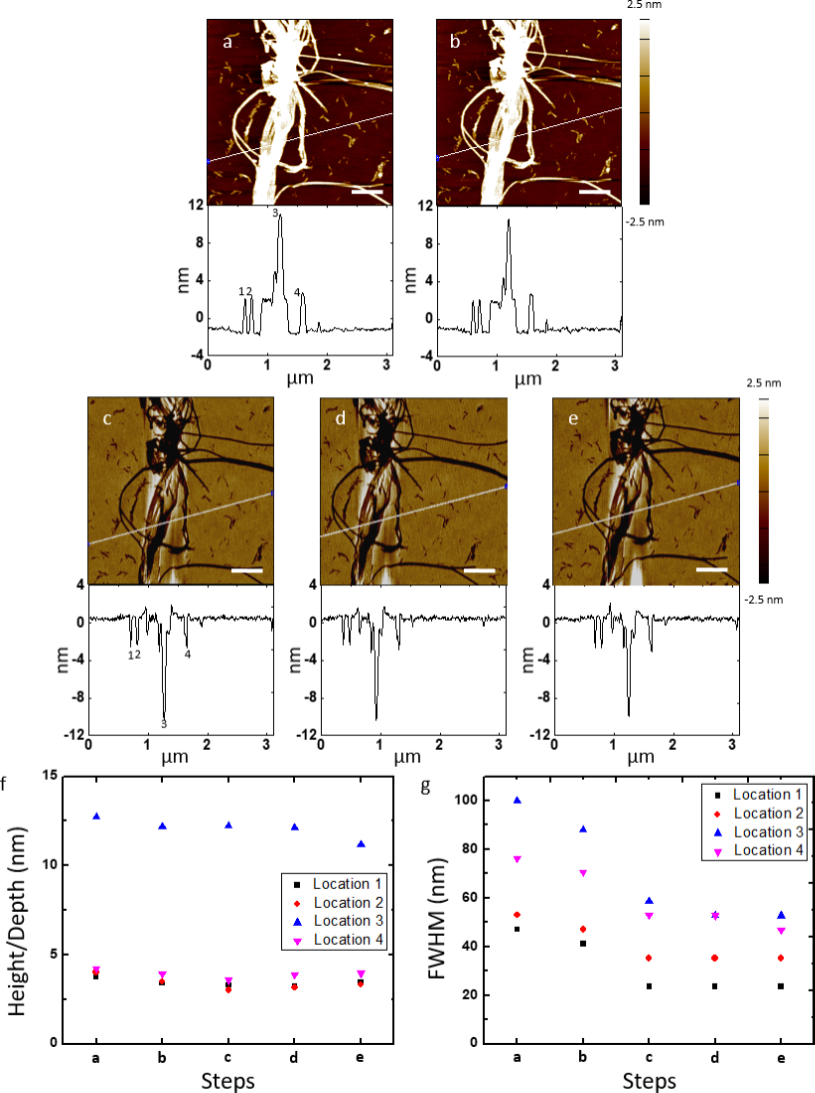
Comparison of features on a DNA nanotube master template with a ca. 2 nm thick Al_2_O_3_ film and a PLLA stamp, and stability of features on a PLLA stamp of the same area. AFM height images and corresponding cross-sectional analysis of DNA nanotubes after (a) deposited on a silicon wafer and (b) 20 cycles of ALD of Al_2_O_3_ (ca. 2 nm of Al_2_O_3_ film), and the negative replicas on a PLLA stamp imaged (c) 1, (d) 8, and (e) 15 days after pattern transfer of the same area. White lines on the AFM images indicate where the cross-sections were determined. (f) Height/depth and (g) FWHM of the DNA nanotubes and their replica trenches in four different locations of the AFM images from (a) to (e). Locations 1, 2, 3, and 4 correspond to 1, 2, 3, and 4 in the cross-sections of the AFM images (a) and (c). Scale bars represent 500 nm. Note: The AFM images from (c) to (e) are mirror-flipped to match the orientations of the AFM images (a) and (b).

We first evaluate the fabrication process using a self-assembled DNA nanotube template. These DNA nanotubes are 30–70 nm in width and up to 60 μm in length [4]. The nanotubes are collapsed after deposition onto a silicon wafer, showing an average height (*n* = 10) of 3.4 ± 0.1 nm by atomic force microscopy (AFM). The surface topography of the DNA nanotube master template before (Figure 2a) and after (Figure 2b) deposition of a ca. 2 nm thick Al_2_O_3_ layer and the corresponding PLLA film (Figure 2c) were characterized by AFM. On the DNA nanotube master template, single DNA nanotubes are observed along with some bundles. After the PLLA stamp was peeled off, the negative replicas of the DNA nanotubes were observed on the polymer stamp, demonstrating a faithful replication process. To quantify the degree of conservation of the surface topography, height/depth and full width at half maximum (FWHM) were measured in four different locations in the AFM images and compared at the same locations throughout the fabrication process (Figure 2f,g). Taking location 1 as an example, the height of the DNA nanotube before (3.73 nm) and after (3.39 nm) the ALD of the Al_2_O_3_ film was in good agreement with the average depth of the trench (3.32 nm, measured three times at location 1 over a 15 day period) on the PLLA stamp. The FWHM of the nanotube (46.99 nm) slightly decreased after the ALD (41.14 nm) but was significantly larger than the average FWHM of the trench (23.50 nm) on the polymer stamp. The decrease of the FWHM after the ALD is suspected to be due to the dehydration of the nanotube during the ALD process and/or the differences in the probe–sample interactions of the individual AFM tips, which can give different measurements of the same sample. We attribute the decrease in the FWHM from the DNA nanotube master template to the PLLA stamp to the AFM probe convolution effect. These results confirm a faithful pattern transfer from the DNA nanotube master template to the PLLA stamp through the ALD of the Al_2_O_3_ layer on the template with high fidelity. Moreover, the patterned PLLA stamp was found to be stable at room temperature. We stored the stamp in a plastic petri dish and imaged it again after 1 week (Figure 2d) and 2 weeks (Figure 2e) at the same location. Both the depth and FWHM of the trenches along with cross-sectional analysis on the PLLA stamp at the four locations remained consistent, demonstrating the long-term stability of the PLLA stamp.

**Figure 3:**
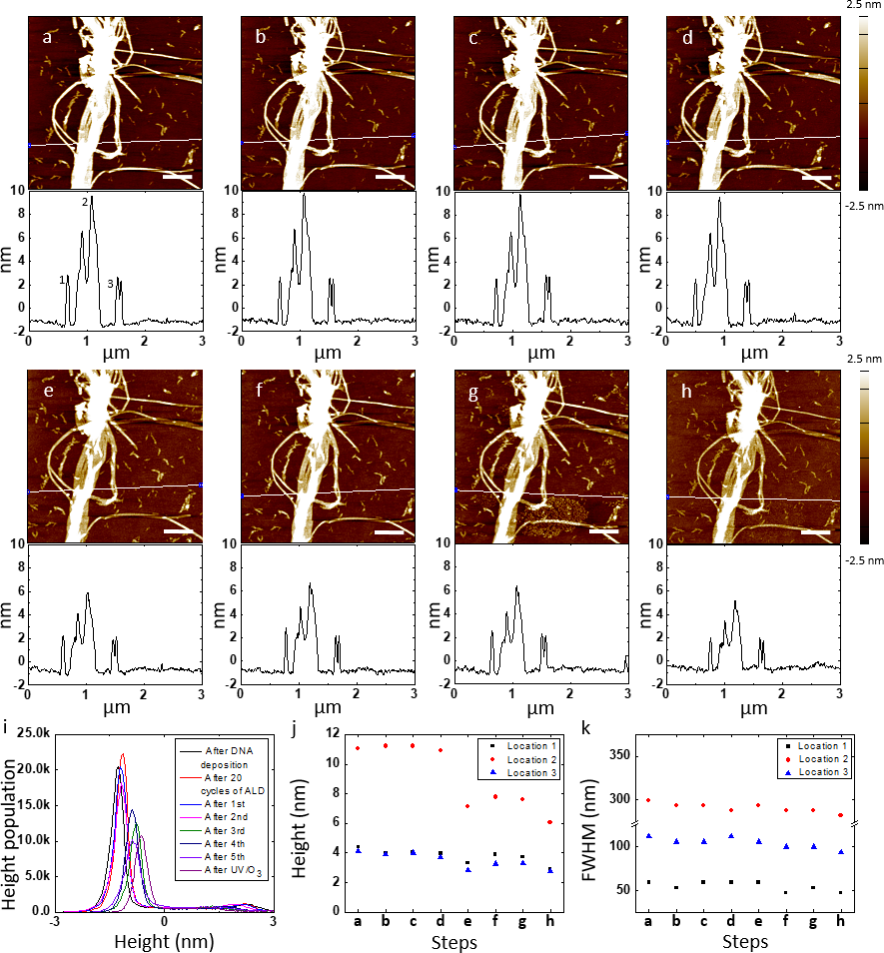
Stability of a DNA nanotube master template with a ca. 2 nm thick Al_2_O_3_ film for multiple pattern transfers to PLLA stamps. AFM height images and corresponding cross-sectional analysis of DNA nanotubes in the same location after (a) deposited on a silicon wafer, (b) 20 cycles of ALD of Al_2_O_3_, (c) 1st, (d) 2nd, (e) 3rd, (f) 4th, and (g) 5th pattern transfer to PLLA stamps, and (h) UV/O_3_ treatment for 1 h and washing with DI water. White lines on the AFM images indicate where the cross-sections were determined. (i) Histograms of the AFM height images from (a) to (h). (j) Height and (k) FWHM of the DNA nanotubes in three different locations of the AFM images from (a) to (h). Locations 1, 2, and 3 correspond to 1, 2, and 3 in the cross-section of the AFM image (a). Scale bars represent 500 nm. Note: The DNA master template was contaminated before the 5th spin coating of PLLA in dichloromethane solution. The AFM images (a) and (b) are also shown in Figure 2. The enlarged version of the histograms in (i) is available in Figure S3 (Supporting Information File 1).

As mentioned earlier, the most critical challenge of using the DNA master template without a protective film is the damage of DNA during the separation of the polymer film from the template [46]. We attribute such damage to the water we used to assist the separation. The DNA nanostructures were still damaged even if we replaced the water with the buffer solution that was used to synthesize and store the DNA nanostructures (Figure S1, Supporting Information File 1). To evaluate the effectiveness of the protective Al_2_O_3_ film on the DNA master template, we imaged the DNA nanostructures in the same location after deposited on a silicon wafer, after 20 cycles of ALD of Al_2_O_3_, and after 1st, 2nd, 3rd, 4th and 5th replication to PLLA stamps (Figure 3a–g and Figure S2a–f, Supporting Information File 1). As the AFM images indicate, the surface morphology of the DNA template was still well maintained after the 1st pattern transfer (Figure 3c and Figure S2b, Supporting Information File 1), showing that the stability of the nanostructures was increased by the ca. 2 nm thick Al_2_O_3_ film. However, as the replication process was repeated another four more times, the overall height of the DNA nanostructures decreased although their shape was unchanged. To highlight the change in the height of the DNA nanostructures, we plot the height distribution of the AFM images in Figure 3i and Figure S3 (Supporting Information File 1). The height difference between the absolute maximum peak (which represents the background silicon wafer) and the next relative maximum peak (which represents the height of the DNA nanotubes) significantly decreased during the 3rd replication process. The height and FWHM with cross-sectional analysis of the DNA template at the three same locations further support the change in the height of the template (Figure 3j,k). The FWHM at all three locations was comparable during the 3rd replication process. The height of the DNA nanotube bundle decreased from 10.90 nm to 7.12 nm, while the height of the single DNA nanotubes decreased from 3.97 nm and 3.70 nm to 3.32 nm and 2.85 nm, respectively. These results indicate that the higher feature (decrease of ca. 35% of its initial height) on the template is less mechanically stable than the lower one (decrease of ca. 15% of its initial height). Along this direction, holes were also formed after the 2nd and 5th pattern transfer to the PLLA stamps, highlighted by the yellow arrows (Figure S2c,f, Supporting Information File 1). The AFM height and phase images with cross-sectional analysis of the hole after the 5th pattern transfer show that the depth of the hole matched well to the thickness of the Al_2_O_3_ layer and the bundle of the DNA nanotubes originally presented in the hole was removed, possibly by the water used during the separation of the stamp (Figure S2h,i, Supporting Information File 1). Overall, the protective 2 nm Al_2_O_3_ layer marginally increases the stability of the DNA nanostructures.

Being able to clean the master template is also important for its repeated use. During the five times of the pattern transfer to the PLLA stamps, the surface of the DNA master template was contaminated with polymer residues (see Figure 3g, lower middle area). To verify whether the polymer residues on the DNA master template can be removed with UV/O_3_ treatment, the template after the 5th replication process was subjected to UV/O_3_ cleaning for an hour, washed with deionized (DI) water, and dried with N_2_ gas (Figure 3h and Figure S2g, Supporting Information File 1). The AFM images before and after the treatment show that the morphology of the DNA template was not altered while the polymer residues were removed. The height difference between the absolute maximum peak and the next relative maximum peak in the histogram of the AFM image, however, significantly decreased from 2.28 nm to 1.69 nm (Figure 3i and S3). The height of the DNA nanotubes at three different locations decreased from 3.72 nm, 7.58 nm, and 3.29 nm to 2.87 nm, 6.07 nm, and 2.78 nm, respectively (Figure 3j). The FWHM at these locations also decreased from 52.88 nm, 287.94 nm, and 99.89 nm to 46.98 nm, 281.87 nm, and 93.96 nm, respectively (Figure 3k). These results suggest that although the UV/O_3_ treatment is able to eliminate the organic residues on the surface of the master template, the DNA nanostructures beneath the 2 nm of Al_2_O_3_ coating are likely damaged by the oxidation by O_3_.

The long-term stability of the ALD-coated template was also studied. We kept the template in a plastic petri dish that was stored in a common lab bench drawer for 40 days. AFM images with corresponding cross-sectional analysis were scanned in the same location of the template at the beginning and the end of this period (Figure S4a,b, Supporting Information File 1). Not surprisingly, the 40 days of aging in air did not alter the surface topography of the DNA nanostructure master template. While the height of the DNA nanotubes at four different locations remained consistent (Figure S4c, Supporting Information File 1), the FWHM at these locations slightly decreased (Figure S4d, Supporting Information File 1). We speculate that the decrease in the FWHM results from the differences between the AFM probe convolution effects of the individual tips because the decreases are similar to the resolution limit of the AFM image (i.e., one or two pixels in the AFM images). At room temperature, solid-state DNA undergoes degradation and/or aggregation within 30 days when it is exposed to atmospheric water and oxygen [47–48]. Compared to DNA, which is a soft material, Al_2_O_3_ is much more stable and robust. Through the conformational coating of Al_2_O_3_, the shelf life of the DNA nanotubes is assumed to be increased while maintaining their morphology longer than the nanotubes without a protective film. Overall, the 20 cycles of ALD of Al_2_O_3_ allow the DNA nanostructure master template to possess enough chemical stability for long-term storage.

**Figure 4:**
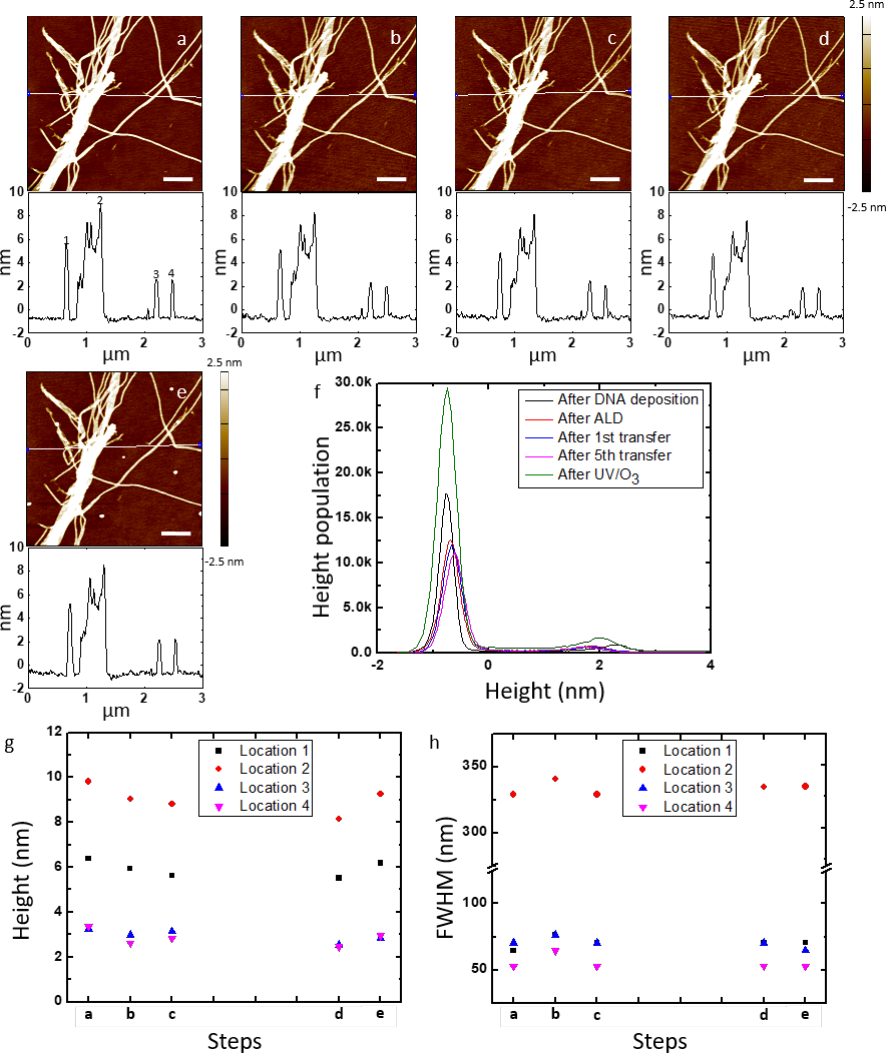
Stability of a DNA nanotube master template with a ca. 5 nm thick Al_2_O_3_ film for multiple pattern transfers to PLLA stamps. AFM height images and corresponding cross-sectional analysis of DNA nanotubes in the same location after (a) deposited on a silicon wafer, (b) 50 cycles of ALD of Al_2_O_3_, (c) 1st and (d) 5th pattern transfer to PLLA stamps, and (e) UV/O_3_ treatment for 1 h and washing with DI water. White lines on the AFM images indicate where the cross-sections were determined. (f) Histograms of the AFM height images from (a) to (e). (g) Height and (h) FWHM of the DNA nanotubes in four different locations of the AFM images from (a) to (e). Locations 1, 2, 3, and 4 correspond to 1, 2, 3, and 4 in the cross-section of the AFM image (a). Scale bars represent 500 nm. Note: The enlarged version of the histograms in (f) is available in Figure S6 (Supporting Information File 1).

**Figure 5:**
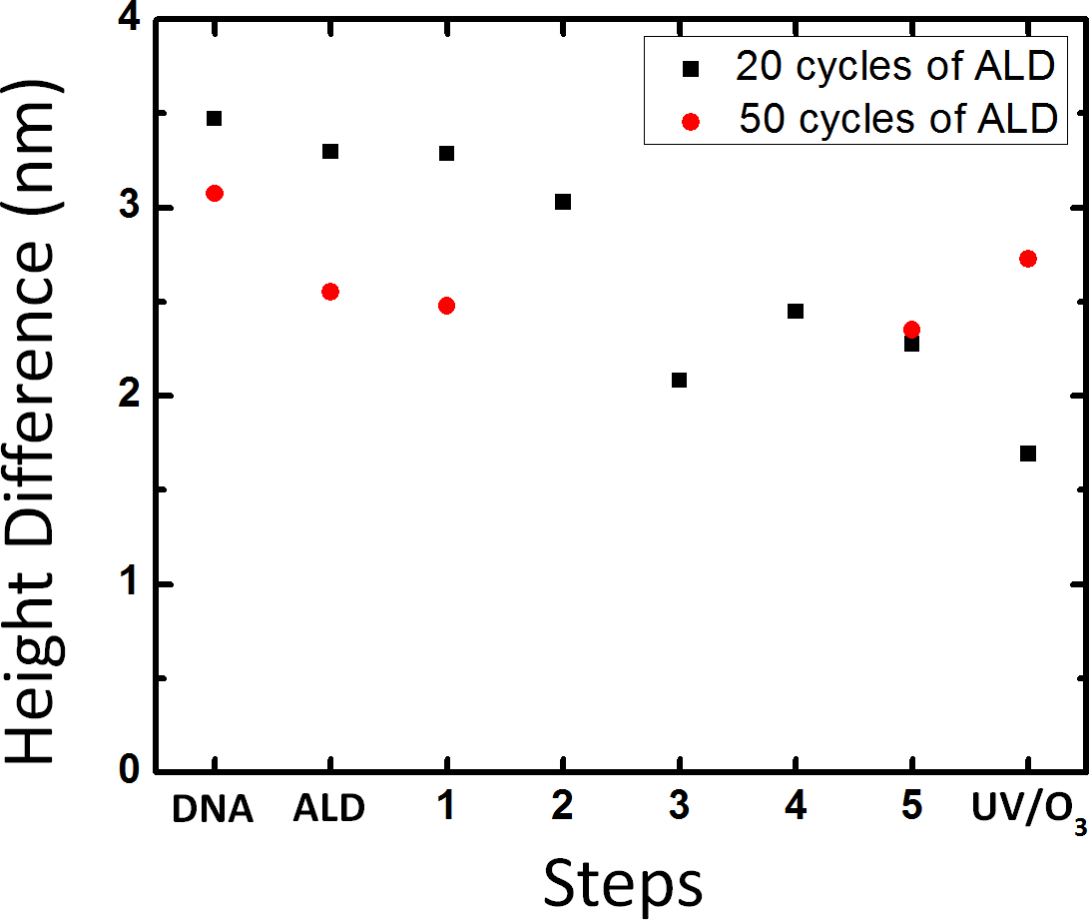
Comparison of the height differences between the maximum peaks of the histograms in Figure 3i and 4f.

The ca. 2 nm thick Al_2_O_3_ layer increased the mechanical stability of the DNA nanotube master template only to a limited extent. To verify whether the mechanical stability of the template can be strengthened with the increased thickness of the Al_2_O_3_ layer while preserving its nanoscale morphology, a ca. 5 nm thick Al_2_O_3_ layer was deposited onto the template, and the reusability and morphology conservation were evaluated. The DNA nanostructures in the same location were scanned with AFM after deposition on a silicon wafer, 50 cycles of ALD of Al_2_O_3_, 1st and 5th replication to PLLA stamps, and exposed to UV/O_3_ treatment, washed with DI water, and dried with N_2_ gas (Figure 4a–e and Figure S5a–c, Supporting Information File 1). Throughout each stage of the fabrication process, we analyzed the height difference between the absolute maximum peak and the next relative maximum peak in the histogram and height and FWHM at four different locations; all these data showed little change throughout the fabrication process (Figure 4f–h and Figure S6, Supporting Information File 1). The ca. 5 nm thick Al_2_O_3_ film is impermeable to O_3_ and protects the underlying DNA nanostructures against the UV/O_3_ oxidation. Also, no holes due to the breakage of the protective Al_2_O_3_ film were found, demonstrating that the both chemical and mechanical stabilities of the DNA nanostructure master template improve with a thicker Al_2_O_3_ layer. The direct comparison of the height differences between the maximum peaks of the histograms of the 20 and 50 cycles of ALD of Al_2_O_3_ through the multiple pattern transfer clearly shows the increased stability of the ca. 5 nm thick Al_2_O_3_ film compared to the ca. 2 nm thick film (Figure 5). We note that the polymer residue was not observed on the surface of the DNA nanotube master template with the ca. 5 nm thick Al_2_O_3_ film even after the 5th replication. The surface roughness of Al_2_O_3_ film grown using ALD slowly increases as the number of cycles goes up [49]. Therefore, it does not cause the reduced polymer adsorption on the 5 nm thick Al_2_O_3_ film. Further study is needed to elucidate the difference between the 2 nm and 5 nm of Al_2_O_3_ films.

**Figure 6:**
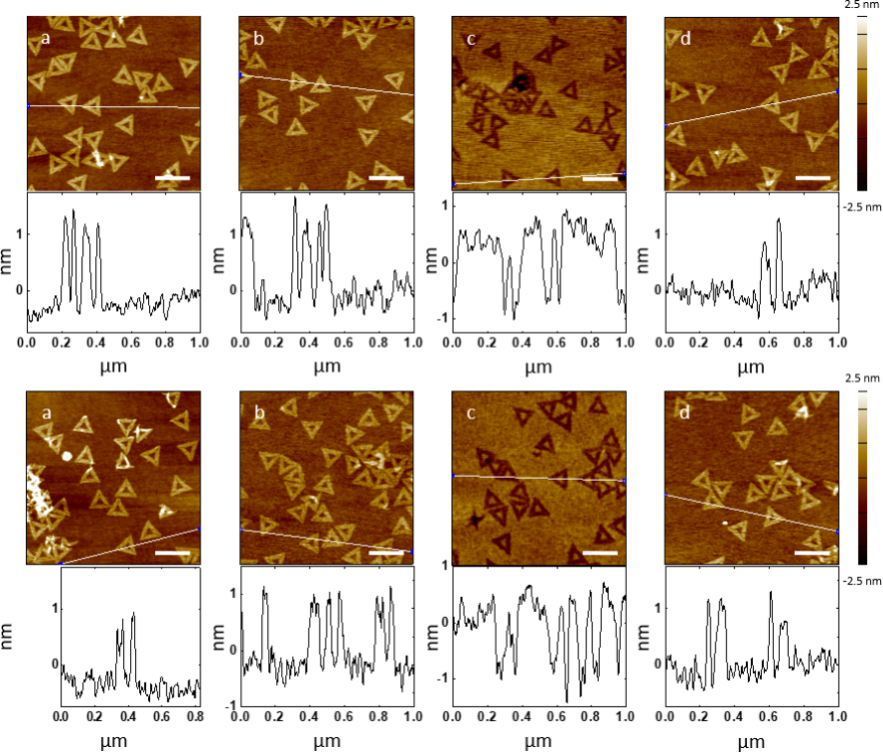
Comparison of features on DNA origami triangle master templates with a ca. 2 nm or a ca. 5 nm thick Al_2_O_3_ layer and PLLA stamps. AFM height images and corresponding cross-sectional analysis of origami triangles after (a) deposited on silicon wafers, (b) 20 cycles (top) or 50 cycles (bottom) of ALD of Al_2_O_3_, and (d) pattern transfer to PLLA stamps, and (c) their negative replicas on the PLLA stamps. White lines on the AFM images indicate where the cross-sections were determined. Scale bars represent 200 nm.

**Figure 7:**
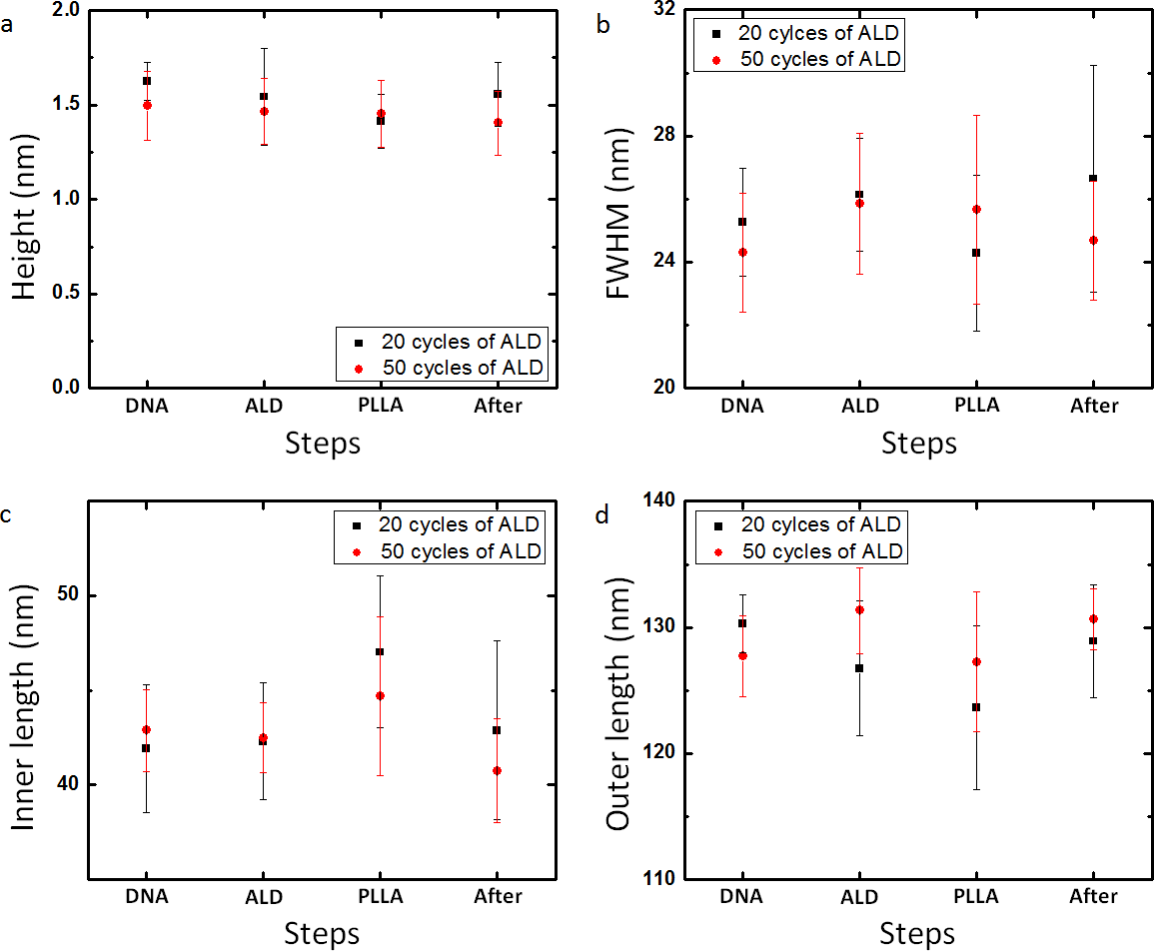
Average (a) height, (b) FWHM, (c) inner length, and (d) outer length (n = 10) of features on DNA origami triangle master templates with a ca. 2 nm or ca. 5 nm of Al_2_O_3_ layer and PLLA stamps at each step of fabrication process, after (DNA) DNA origami triangles were deposited on a silicon wafers, (ALD) ALD of Al_2_O_3_, and (After) pattern transfer to PLLA stamps, and (PLLA) their negative replicas on the PLLA stamps.

The DNA nanotubes tested above are simple one-dimensional linear structures. To evaluate the effectiveness of a protective conformational coating on complex patterns, DNA origami triangle nanostructures were employed as the master templates for the pattern transfer to the PLLA stamp. The DNA origami triangle is a single layer of DNA double strands and has a theoretical height of 2 nm (Figure S7, Supporting Information File 1) [8]. The triangle consists of three trapezoidal domains formed by folding an M13mp18 scaffold strand with short synthetic staple strands. Among the three trapezoidal domains, one has a dangling loop. These domains are further connected to each other by bridging the edges of the domains with the staple strands. There are three holes at each of the vertex and one large triangular hole in the center of the DNA origami triangle. AFM images show that the three holes at the vertex, the central triangular hole, and the dangling loop were clearly visible before and after ALD, and after replication process with both ca. 2 nm and ca. 5 nm thick Al_2_O_3_ layers (Figure 6a,b,d). Through these steps, the three holes at the vertex were frequently seen as a linear gap and the depth of the holes or the linear gap was much smaller than the height of the nanostructures due to the limited resolution of the AFM images. The vertex with the holes or the linear gap was highlighted by the blue dots (Figure S8d and Figure S9d, Supporting Information File 1). The dangling loop was also highlighted by the yellow arrows (Figure S8a,d and Figure S9a,d, Supporting Information File 1). The loop might not be seen in some DNA origami triangles if the loops were folded above or beneath the DNA structures. According to the cross-sectional analysis of the AFM images, the average height, FWHM, inner length and outer length of the DNA origami triangles remained comparable throughout the replication process including the ALD (Figure 7). All these results prove that the protective Al_2_O_3_ film successfully preserves the surface morphology of the complex DNA origami triangle nanostructures.

After the replication process, triangular trenches resembling the shape of the DNA origami triangles were formed on the PLLA films (Figure 6c). Compared to the dimensions of the DNA triangles with the protective layers on the templates, the average depth of the trenches remained consistent with the average height of the triangles (Figure 7a). Due to the AFM probe convolution, however, the average outer length (the edge length of the trench measured outside of the triangle) and FWHM of the triangular trenches decreased and the average inner length of the trenches increased (Figure 7b–d). Both the patterns corresponding to the dangling loop and the three holes at the vertex were transferred to the PLLA stamps, but they were difficult to find in the trenches compared to the original features on the templates (Figure S8b,c,e and Figure S9b,c,e, Supporting Information File 1). The parts of the trench responsible for the three holes at the vertex and the dangling loop were also highlighted by the blue dots and the yellow arrows, respectively (Figure S8c,e and Figure S9c,e, Supporting Information File 1). On the PLLA stamps, the holes or the linear gap between the trapezoidal domains of the DNA origami triangles are replicated as a small bump at the vertex of the triangular trenches. The height of the bump, however, never reaches the height of the DNA origami triangles and the bump was frequently not observed in some trenches, as the bump on the PLLA stamp peeled off from the DNA origami triangle master template without the protective film. We attribute these observations to the mechanical instability of the bumps during the scanning with AFM and/or the intrinsic limitation of the resolution of the pattern transfer [46]. In the latter case, the large PLLA molecule may not be able to completely fill the nanometer-sized holes in the DNA origami triangles during the spin-coating process. A decrease in the feature size of the DNA nanostructure appears to result in height decrease and/or lost features in the polymer stamp. Overall, the PLLA film is capable of replicating the overall features of the complex DNA origami triangles with high fidelity and the local features below ca. 5 nm only to some extent even with the presence of the protective ca. 2 nm or ca. 5 nm thick Al_2_O_3_ layers.

Finally, we investigated how the surface morphology of the DNA nanostructures was influenced as the thickness of the protective Al_2_O_3_ film was further increased. We coated both the DNA nanotube (Figure S10a,b, Supporting Information File 1) and the DNA origami triangle (Figure S10c,d, Supporting Information File 1) master templates with ca. 20 nm thick Al_2_O_3_ layers and compared their AFM images before (Figure S10a,c, Supporting Information File 1) and after (Figure S10b,d, Supporting Information File 1) 200 cycles of ALD. With the ca. 20 nm thick Al_2_O_3_ film, the DNA nanotubes were still visible and the FWHM stayed consistent (Figure S10b,f, Supporting Information File 1). The height of the DNA nanotubes, however, considerably decreased from 3.83 nm, 9.36 nm, 3.85 nm, and 3.94 nm to 1.54 nm, 2.81 nm, 1.66 nm, and 1.71 nm, respectively (Figure S10e, Supporting Information File 1). In case of the DNA origami triangles, the DNA nanostructures with the average height of 1.68 nm (*n* = 10) were barely seen and the height proﬁle along the individual DNA triangles also showed the signiﬁcant increase of roughness (Figure S10d, Supporting Information File 1). These results indicate that there is a limit to the thickness of the protective Al_2_O_3_ film deposited by ALD to maintain the nanoscale feature of the DNA nanostructure on the template.

## Conclusion

We have reported a method to increase the stability of DNA nanostructure master templates through the conformal growth of an inorganic oxide film by ALD and demonstrated its usefulness in soft lithography patterning of polymer films. DNA nanotubes and origami triangles with Al_2_O_3_ films of ca. 2 nm, ca. 5 nm or ca. 20 nm thickness have been tested as the master templates to imprint their nanoscale features to PLLA films. As the thickness of the Al_2_O_3_ coating grows, the mechanical and/or chemical stability increases while some of the nanoscale features of the DNA nanostructures are lost. Based on our results, the conformational coating of the ca. 5 nm thick Al_2_O_3_ layer to the DNA nanostructures provides a good compromise between increasing the stability and maintaining the nanoscale feature of the master template for repeated use in soft lithography. In addition, the ca. 5 nm thick Al_2_O_3_ layer offered good protection to the underlying DNA nanostructures from exposure to UV/O_3_. Although our study focused on the ALD of Al_2_O_3_, other metals, metal oxides, or inorganic oxides can also be used as long as they can be conformally coated at a temperature below 250 °C. Above 250 °C, the degradation of DNA nanostructures deposited onto silicon wafers starts to occur although the decomposition residue may still maintain their nanoscale features [50–51]. The conformal protective film significantly improves the chemical and mechanical stabilities of DNA nanostructures, allowing them to be used in environments that are incompatible with pristine DNA nanostructures.

## Experimental

### Materials

Silicon wafers [Si(110), with native oxide] and M13mp18 scaffold strands for DNA origami triangles were purchased from University Wafers (South Boston, MA, USA) and Bayou Biolabs (Metairie, LA, USA), respectively. Staple strands for the DNA origami triangles and strands for DNA nanotubes were synthesized by Integrated DNA Technologies (Coralville, IA, USA). 2-Amino-2-(hydroxymethyl)-1,3-propanediol (Tris), ethylenediaminetetraacetic acid (EDTA), magnesium acetate tetrahydrate, sulfuric acid, hydrogen peroxide solution (30% H_2_O_2_), and poly(L-lactide) were purchased from Sigma-Aldrich (St. Louis, MO, USA). Acetic acid (glacial), dichloromethane, and ethanol were purchased from Fisher Scientific (Fair Lawn, NJ, USA), Acros Organics (Fair Lawn, NJ, USA), and Decon Laboratories, Inc. (King of Prussia, PA, USA), respectively. PDMS backing stamp was fabricated with Sylgard 184 silicone elastomer kit (Dow Corning, Midland, MI, USA). All materials were used as received. High-purity water (18.3 MΩ) was used throughout the entire experiment by using a Barnstead MicroPure Standard water purification system (Thermo Scientific, Waltham, MA, USA).

### Preparation of a silicon wafer

A silicon wafer with a native oxide layer was cleaned by hot piranha solution [7:3 (v/v) concentrated H_2_SO_4_/30% H_2_O_2_]. After H_2_O_2_ was slowly added to concentrated H_2_SO_4_ in a glass petri dish containing the silicon wafer, a glass cover was placed and a heating plate was set to 40 °C. After 20 min, the heating plate was turned off and the piranha solution was allowed to cool down for an additional 10 min. The wafer was thoroughly washed with deionized water and dried with N_2_ gas. Warning: Piranha solution is a strong oxidizing reagent and reacts violently with organic materials. All work should be handled in a fume hood with extra caution. Proper protective equipment is required.

### Preparation and deposition of DNA nanotubes on a silicon wafer

The synthesis and assembly of DNA nanotubes followed a previously published procedure [4]. Single strands of DNA nanotubes were diluted to a final concentration of 1 μM in 10 × TAE/Mg^2+^ buffer (125 mM Mg^2+^). The DNA single strand solution was slowly cooled from 95 to 23 °C over 2 days and stored at 4 °C overnight. Annealed DNA nanotubes were assembled on a clean silicon wafer by incubating the DNA nanotube solution on the wafer for a minimum of 15 min in a humid chamber to minimize the evaporation of the buffer solution. The sample was dried with N_2_ gas, immersed in ethanol/water [9:1 (v/v)] solution for 10 s to remove ionic salt residue from the buffer solution, and re-dried with N_2_ gas. After the deposition, the DNA nanotube master template was processed with ALD of Al_2_O_3_ within 24 h.

### Preparation and deposition of DNA origami triangles on a silicon wafer

DNA origami triangles were synthesized and assembled following a formerly reported method [8]. M13mp18 scaffold strands (8.6 μL, 1.6 nM) were thoroughly mixed with a desired set of synthetic 232 short staple strands (15 μL, 16 nM), deionized water (77 μL), and TAE/Mg^2+^ buffer solution (181 μL). The buffer solution was prepared by dissolving Trizma base (40 mM), EDTA (2 mM), acetic acid (2mM), and magnesium acetate tetrahydrate (150 mM) in deionized water and further diluting the solution to make the final concentration of magnesium ions 12.5 mM. The DNA solution was cooled from 95 to 20 °C at a rate of 1 °C/min. After the annealing, excess staple strands were removed by purifying 140 μL of the DNA origami triangle solution using 500–600 μL of the TAE/Mg^2+^ buffer in a Microcon YM-100 100 kDa MW centrifuge filter (Millipore, Billerica, MA, USA) on a single-speed benchtop Galaxy Ministar microcentrifuge (VWR, Radnor, PA, USA) until the final volume of the DNA origami triangle solution was the same as before the purification. The rinsing process was repeated two more times.

DNA origami triangles were assembled on a clean silicon wafer by incubating the purified DNA solution on the wafer for a minimum of 15 min in a humid chamber to minimize the evaporation of the buffer solution. The sample was dried with N_2_ gas, immersed in ethanol/water [9:1 (v/v)] solution for 3 s to remove ionic salt residue from the buffer solution, and re-dried with N_2_ gas. After the deposition, the DNA origami triangle master template was processed with ALD of Al_2_O_3_ within 24 h.

### Atomic layer deposition (ALD) of Al_2_O_3_ as a protective inorganic film on a DNA master template

ALD of Al_2_O_3_ on a DNA/SiO_2_ substrate followed a previously published method [36]. ALD was conducted using a Fiji ALD system by Norman Gottron in Nanofabrication Facility at Carnegie Mellon University (Ultratech/CNT, Waltham, MA, USA). Chamber and substrate heaters were set to 200 °C. Total Ar gas flow was at 260 sccm and 200 mTorr. Trimethylaluminum (TMA) and H_2_O were used as precursors and one ALD cycle consisted of a 0.06 s long TMA pulse, a 10 s long interval, a 0.06 s long H_2_O pulse and a 10 s long interval. Deposition was looped 20 times, 50 times, and 200 times for the 2 nm, 5 nm, and 20 nm preset deposition thickness of the oxide films, respectively.

### Preparation of a PDMS backing film

PDMS precursor was mixed with curing agent at a 9:1 (v/v) ratio. The prepolymer mixture was vigorously stirred by hand at least for 5 min and degassed in a vacuum desiccator. The mixture was poured over a piranha cleaned silicon wafer. The wafer with the mixture was placed in the vacuum desiccator for further degassing. The PDMS prepolymer on the silicon wafer was cured for 1 hour at 60 °C. The thickness of the resulting PDMS layer was 1–2 mm.

### Fabrication of a PLLA stamp using a DNA nanostructure master template with a protective Al_2_O_3_ film

PLLA stamps were fabricated following our previously demonstrated procedure [33]. PLLA in dichloromethane solution (3 wt %) was spin-coated four times onto a DNA nanostructure master template with an Al_2_O_3_ film at 4000 rpm for 30 s. Around the border of PLLA film surface, the surface perimeter of the PLLA film with the widths of ca. 1 mm were scraped off to expose the underlying template. A PDMS stamp with a thickness of ca. 1–2 mm was placed on top of the PLLA film as a backing stamp. Droplets of water were added to the exposed edges of the template. If the water droplets filled out the interface between the PLLA film and the PDMS backing stamp, they were removed using a paper wiper to increase the adhesion between the polymer film and the backing stamp. After a minute, the PLLA/PDMS film was peeled off and the surface of the PLLA film was gently dried with N_2_.

### UV/Ozone treatment

A DNA nanotube master template with an Al_2_O_3_ film was placed in a PSD Pro 4 Digital UV Ozone Cleaner (Novascan Technologies, Inc., Ames, IA, USA). Before UV irradiation, the chamber was flushed with O_2_ for 3 min, and the sample was subjected to UV/O_3_ treatment for 60 min at room temperature.

### Characterization methods

**Ellipsometry**: The experimental thickness of an Al_2_O_3_ ﬁlm was measured by an Alpha-Spectroscopic Ellipsometer with Complete Ease Software using Cauchy model (JA Woollam Co., Lincoln, NE, USA). Duration time was “Standard” and the measurement angle was 70°. For each sample, the average thickness of the Al_2_O_3_ layer was obtained by measuring the thickness with MSE values below 5 at five different locations.

**Atomic Force Microscopy:** The surface morphologies of a DNA nanostructure master template and a PLLA stamp at each step of fabrication process were imaged using tapping-mode on an MFP-3D atomic force microscope with RTESPA-300, NSC15/Al BS, or SSS-FMR-SPL AFM probes in air at room temperature (Oxford Instruments Asylum Research, Inc., Santa Barbara, CA, USA). The RTESPA-300 (300 kHz, 40 N/m) and NSC15/AL BS (325 kHz, 40 N/m) AFM probes were purchased from Bruker (Camarillo, CA, USA) and MikroMasch (Lady’s Island, SC, USA), respectively, and used to scan the DNA nanotube master templates and the corresponding PLLA stamps. The SSS-FMR-SPL AFM probe (75 kHz, 2.8 N/m) was purchased from NanoAndMore USA (Watsonville, CA, USA) and was used to scan the DNA origami triangle master templates and the corresponding PLLA stamps.

## Supporting Information

File 1 Additional experimental data.
