# Supplementary material for: Increasing the stability of DNA nanostructure templates by atomic layer deposition of Al2O3 and its application in imprinting lithography
Source: Beilstein J Nanotechnol. 2017 Nov 9;8:2363–75. doi: 10.3762/bjnano.8.236 (PMC5687006; doi:10.3762/bjnano.8.236)
Supplement: File 1 — Additional experimental data. [file Beilstein_J_Nanotechnol-08-2363-s001.pdf]

# Supporting Information

for

# Increasing the stability of DNA nanostructure templates by atomic layer deposition of $Al_2O_3$ and its application in imprinting lithography

Hyojeong Kim, Kristin Arbutina, Anqin Xu and Haitao Liu*

Address: Department of Chemistry, University of Pittsburgh, 219 Parkman Avenue, Pittsburgh, Pennsylvania 15260, United States of America

Email: Haitao Liu* - hliu@pitt.edu

*Corresponding author

# Additional experimental data

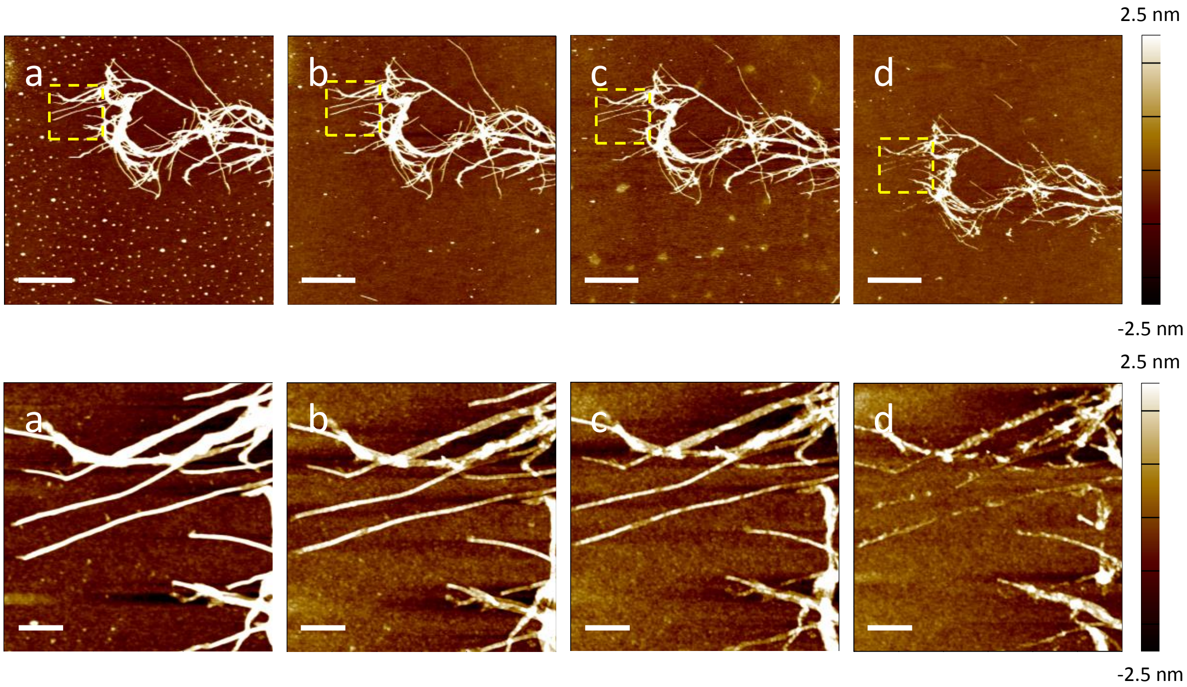


**Figure S1:** Stability of a DNA nanotube master template without a protective inorganic oxide film during multiple pattern replication process using 1× TAE/$Mg^{2+}$ buffer. AFM height images of DNA nanotubes in the same location of a silicon wafer (a) before and after (b) 1st, (c) 2nd, and (d) 3rd pattern transfers to PLLA stamps using 1× TAE/$Mg^{2+}$ buffer. The bottom row contains the zoomed-in views of the areas in the yellow dashed boxes in the top row. Scale bars represent 3 µm (top row) or 500 nm (bottom row).

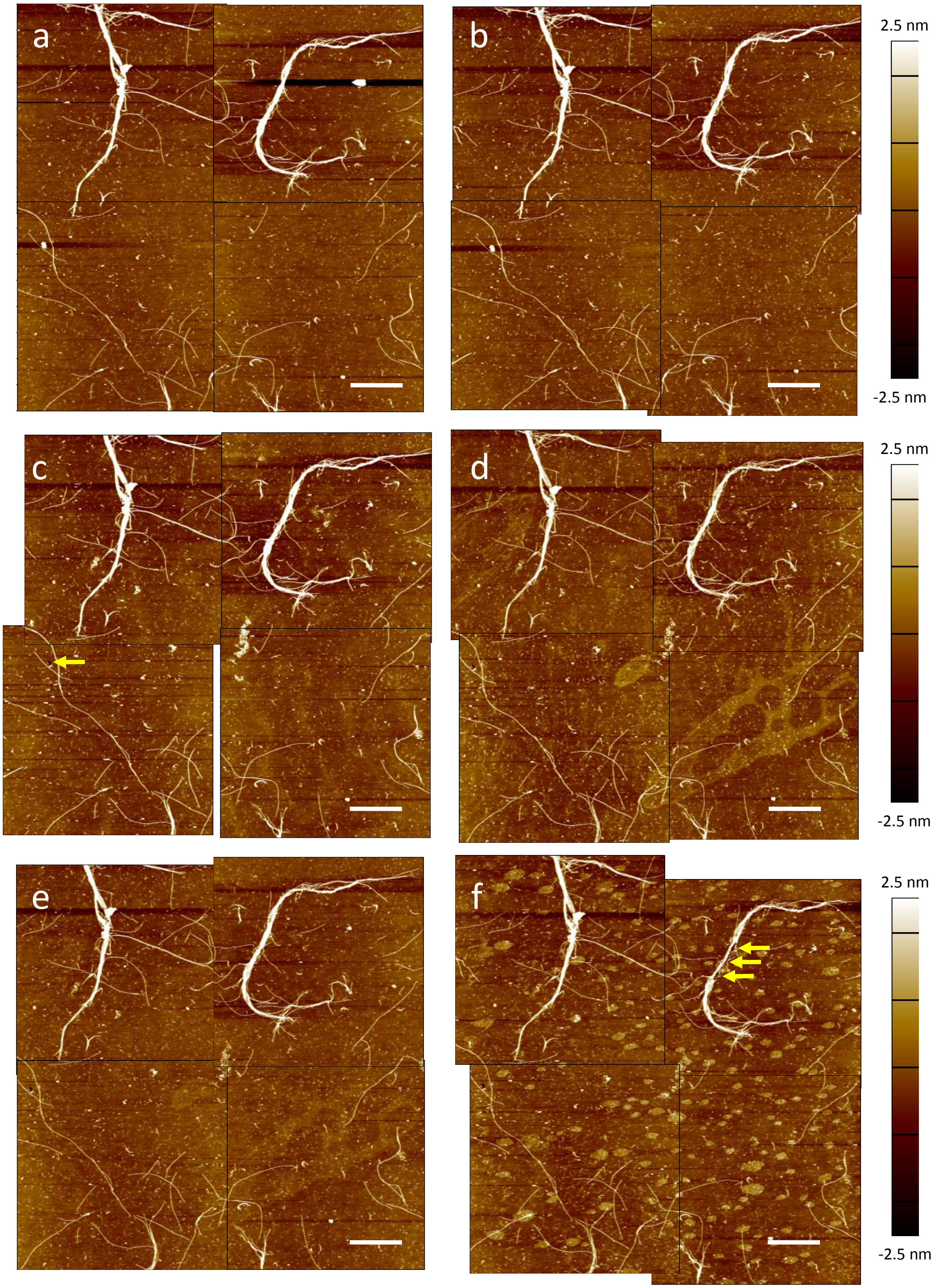
a
b
2.5 nm
-2.5 nm
c
d
2.5 nm
-2.5 nm
e
f
2.5 nm
-2.5 nm

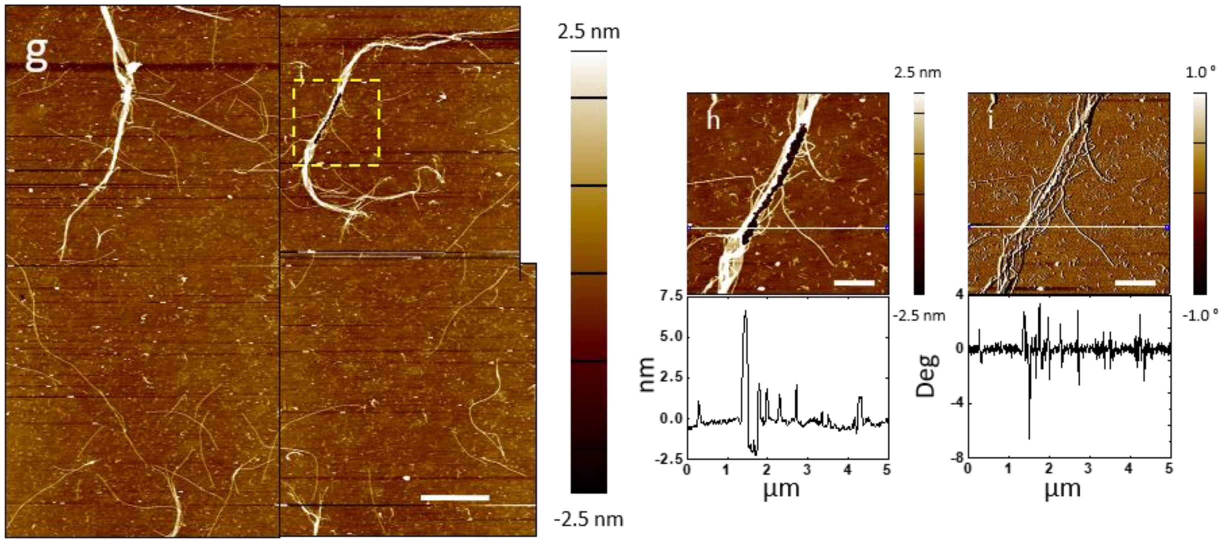


**Figure S2:** Stability of a DNA nanotube master template with a ca. 2 nm thick $Al_2O_3$ film for multiple pattern transfers to PLLA stamps over an area of ca. 30 × 30 µm$^2$. AFM height images of DNA nanotubes in the same location of a silicon wafer after (a) 20 cycles of ALD of $Al_2O_3$, (b) 1st, (c) 2nd, (d) 3rd, (e) 4th, and (f) 5th pattern transfer to PLLA stamps, and (g) UV/$O_3$ treatment for 1 h and washing with DI water. Holes formed by breakage of the $Al_2O_3$ film are indicated by the yellow arrows. AFM (h) height and (i) phase images and corresponding cross-sectional analysis of the area in the yellow dashed box in (g). White lines on the AFM images indicate where the cross-sections were determined. Scale bars represent 4 µm (a–g) or 1 µm (h and i). Note: The DNA master template was contaminated before the 5th spin coating of PLLA in dichloromethane solution.

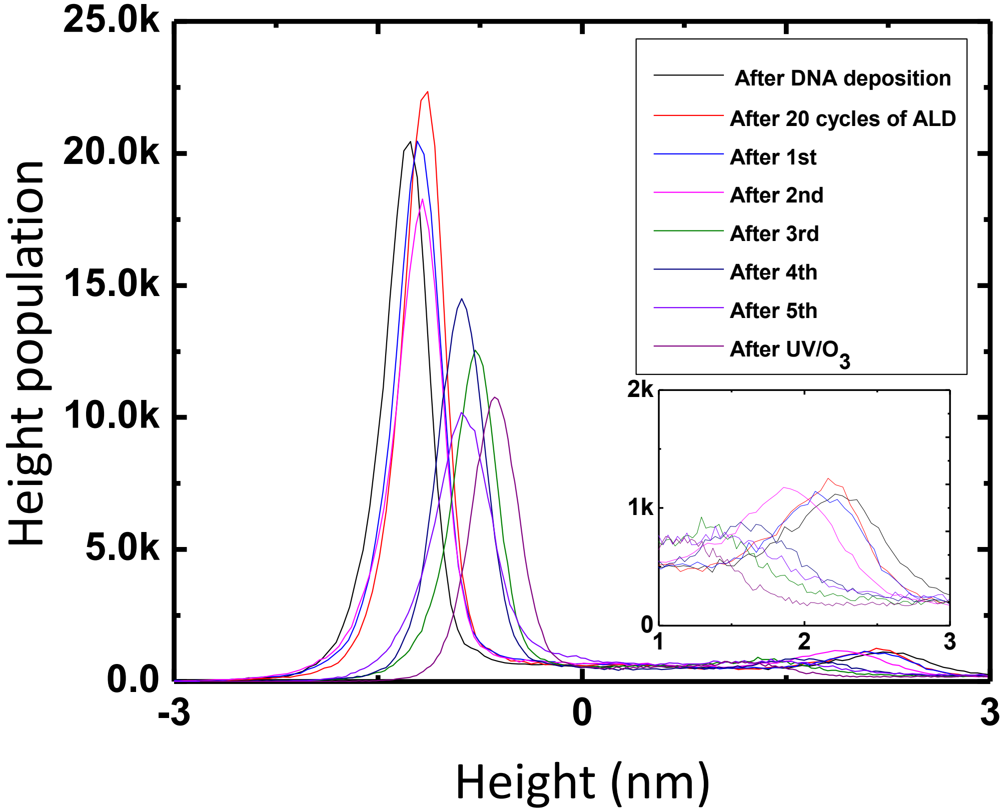


**Figure S3:** Histograms of the AFM height images from Figure 3a to h, the enlarged version of Figure 3i. Height populations from height of 1 nm to 3 nm are further enlarged in the inset.

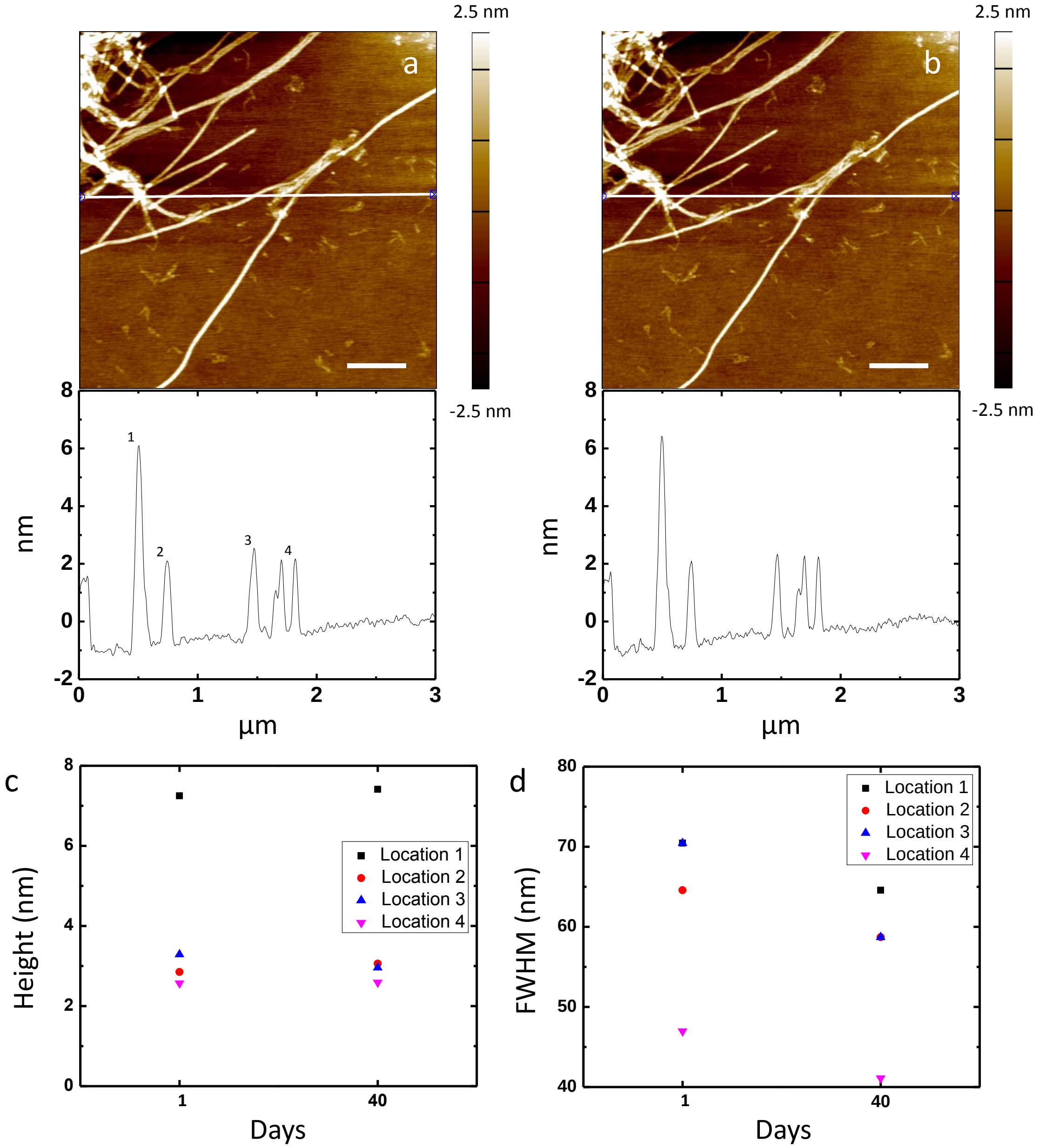


**Figure S4:** Long-term stability of a DNA nanotube master template with a ca. 2 nm thick $Al_2O_3$ film. AFM height images and corresponding cross-sectional analysis of DNA nanotubes in the same location of a silicon wafer at the (a) beginning and (b) end of 40 day period. White lines on the AFM images indicate where the cross-sections were determined. (c) Height and (d) FWHM of the DNA nanotubes in four different locations of the AFM images (a) and (b). Locations 1, 2, 3, and 4 correspond to 1, 2, 3, and 4 in the cross-section of the AFM image (a). Scale bars represent 500 nm.

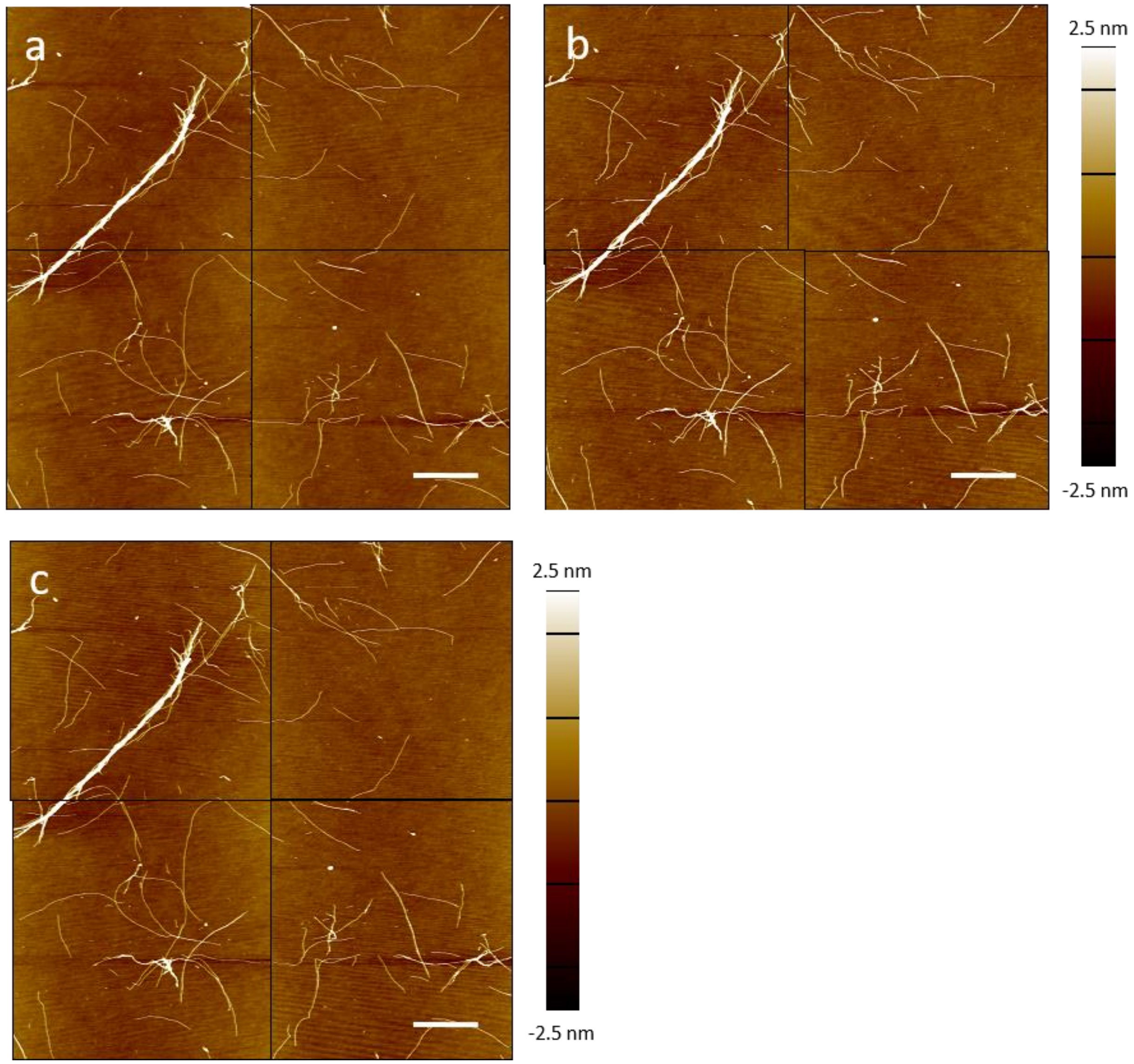


**Figure S5:** Stability of a DNA nanotube master template with a ca. 5 nm thick $Al_2O_3$ film for multiple pattern transfers to PLLA stamps over an area of ca. 30 × 30 $\mu m^2$. AFM height images of DNA nanotubes in the same location of a silicon wafer after (a) 50 cycles of ALD of $Al_2O_3$ and (b) 1st and (c) 5th pattern transfer to PLLA stamps. Scale bars represent 4 µm.

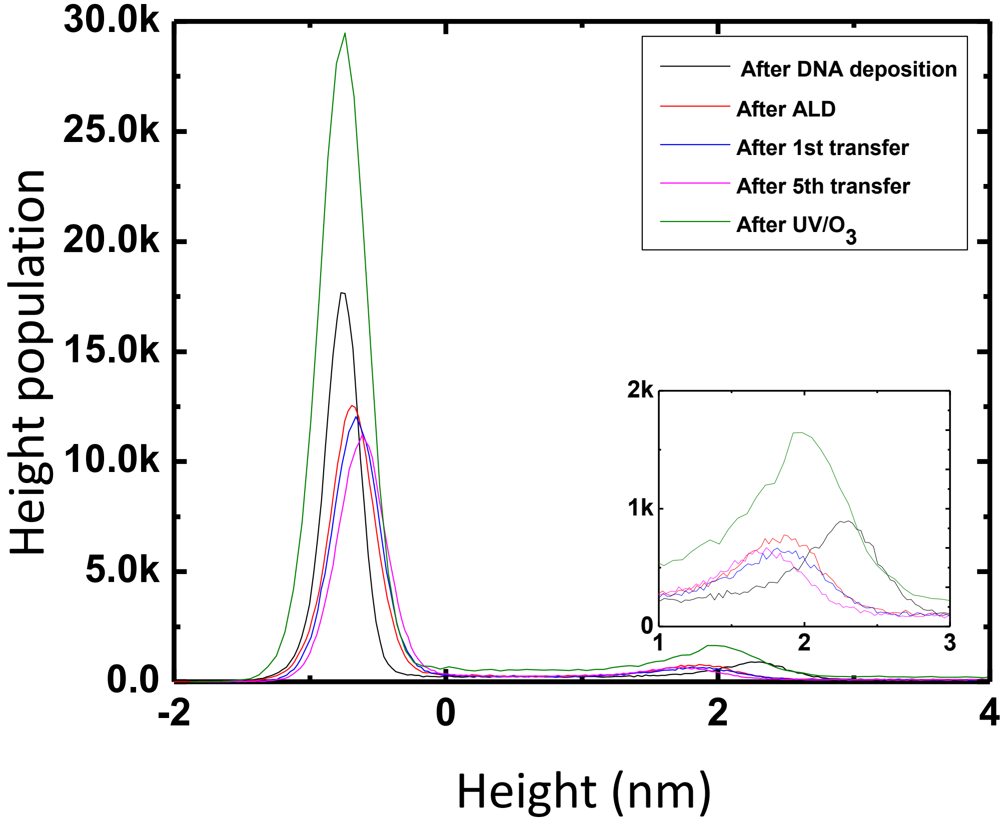


**Figure S6:** Histograms of the AFM height images from Figure 4a–e, the enlarged version of Figure 4f. Height populations from height of 1 nm to 3 nm are further enlarged in the inset.

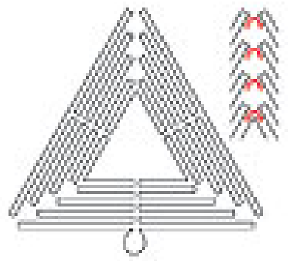

**Figure S7:** Folding path of M13mp18 scaffold strand in DNA origami triangle. Red lines represent synthetic staple strands bridging trapezoidal sides. Reprinted with permission from [1], copyright 2006 Macmillan Publishers Ltd.

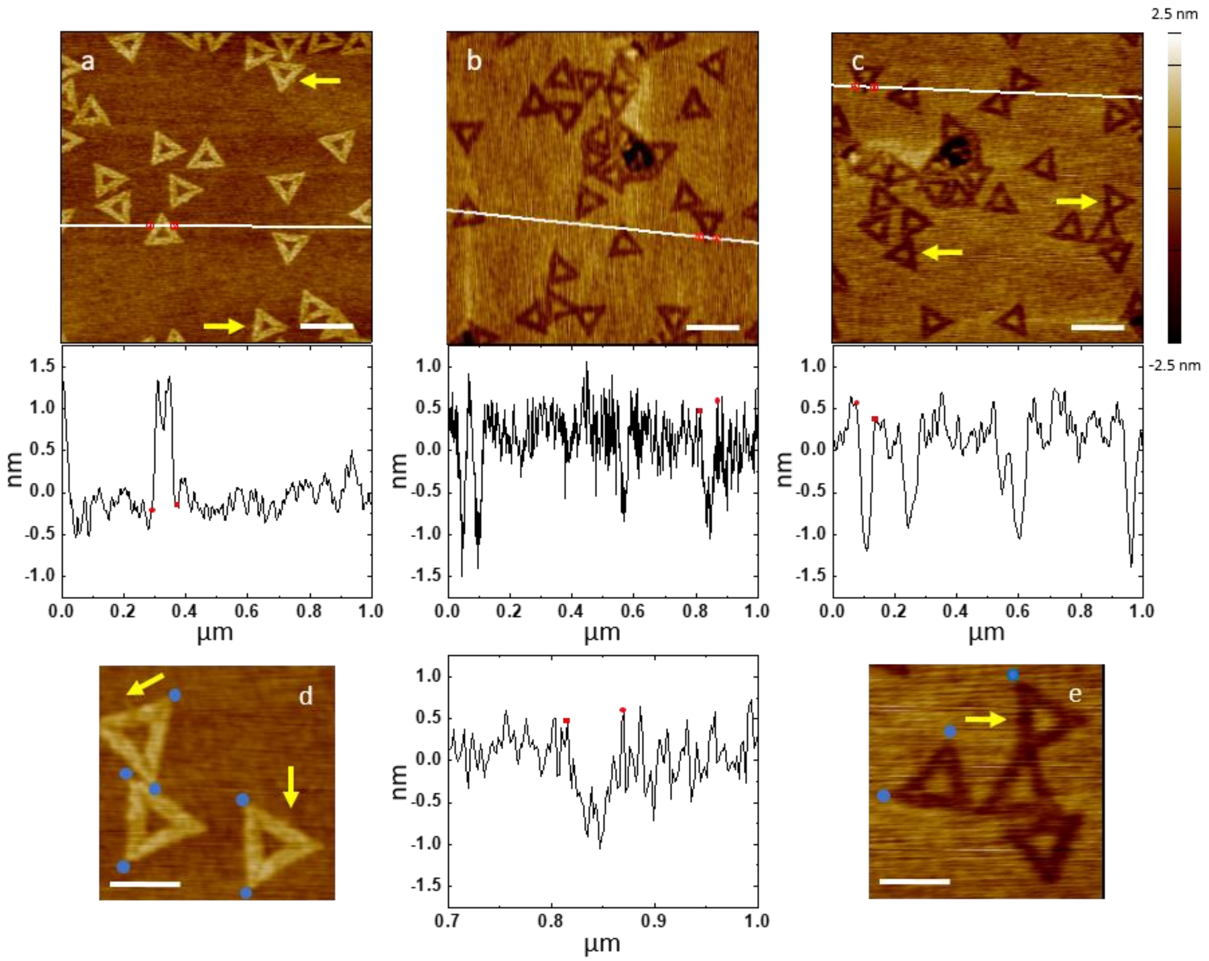


**Figure S8:** Analysis of the dangling loops and vertices of a DNA origami triangle master template with a ca. 2 nm thick $Al_2O_3$ film and a PLLA stamp. AFM height images and corresponding cross-sectional analysis of (a) DNA origami triangles deposited on a silica wafer after 20 cycles of ALD of $Al_2O_3$ and (b,c) their negative replicas on a PLLA stamp. White lines on the AFM images indicate where the cross-sections were determined. The dangling loops are indicated by the yellow arrows. Red cursors in the AFM images and corresponding cross-sections define the outer sides of the vertices that are measured. In (b), the bump appears in the zoomed-in curve valley of the cross-section (bottom), confirming the presence of the bump at the vertex. In (c), the bump does not appear at the curve valley, indicating that no bump exists in the vertex. Zoom-in AFM images of (d) DNA origami triangles and (e) their negative replicas. The dangling loops and vertices with the holes/bumps are indicated by the yellow arrows and the blue dots, respectively. Scale bars represent 200 nm (a–c) and 100 nm (d,e). Note: The AFM images (b) and (c) are identical. The AFM image (b) is rotated 90° clockwise to match the orientation of its corresponding cross-section. The AFM images from (a) to (c) are also shown in Figure 6.

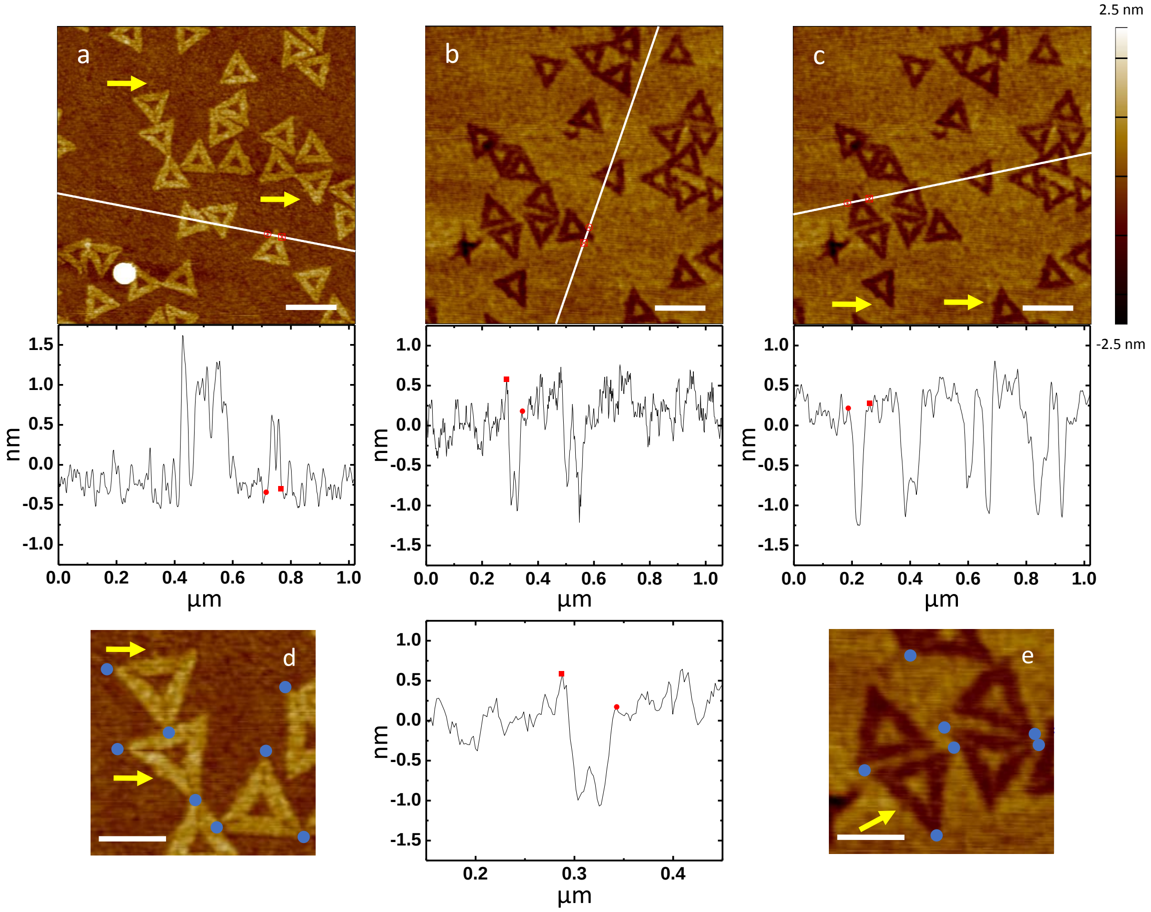


**Figure S9:** Analysis of the dangling loops and vertices of a DNA origami triangle master template with a ca. 5 nm thick $Al_2O_3$ film and a PLLA stamp. AFM height images and corresponding cross-sectional analysis of (a) DNA origami triangles deposited on a silica wafer after 50 cycles of ALD of $Al_2O_3$ and (b,c) their negative replicas on a PLLA stamp. White lines on the AFM images indicate where the cross-sections were determined. The dangling loops are indicated by the yellow arrows. Red cursors in the AFM images and corresponding cross-sections define the outer sides of the vertices that are measured. In (b), the bump appears in the zoomed-in curve valley of the cross-section (bottom), confirming the presence of the bump at the vertex. In (c), the bump does not appear at the curve valley, indicating that no bump exists in the vertex. Zoom-in AFM images of (d) DNA origami triangles and (e) their negative replicas. The dangling loops and vertices with the holes/bumps are indicated by the yellow arrows and the blue dots, respectively. Scale bars represent 200 nm (a–c) and 100 nm (d,e). Note: The AFM images (b) and (c) are identical. The AFM image (b) is rotated 90° clockwise to match the orientation of its corresponding cross-section. The AFM images (b) and (c) are also shown in Figure 6.

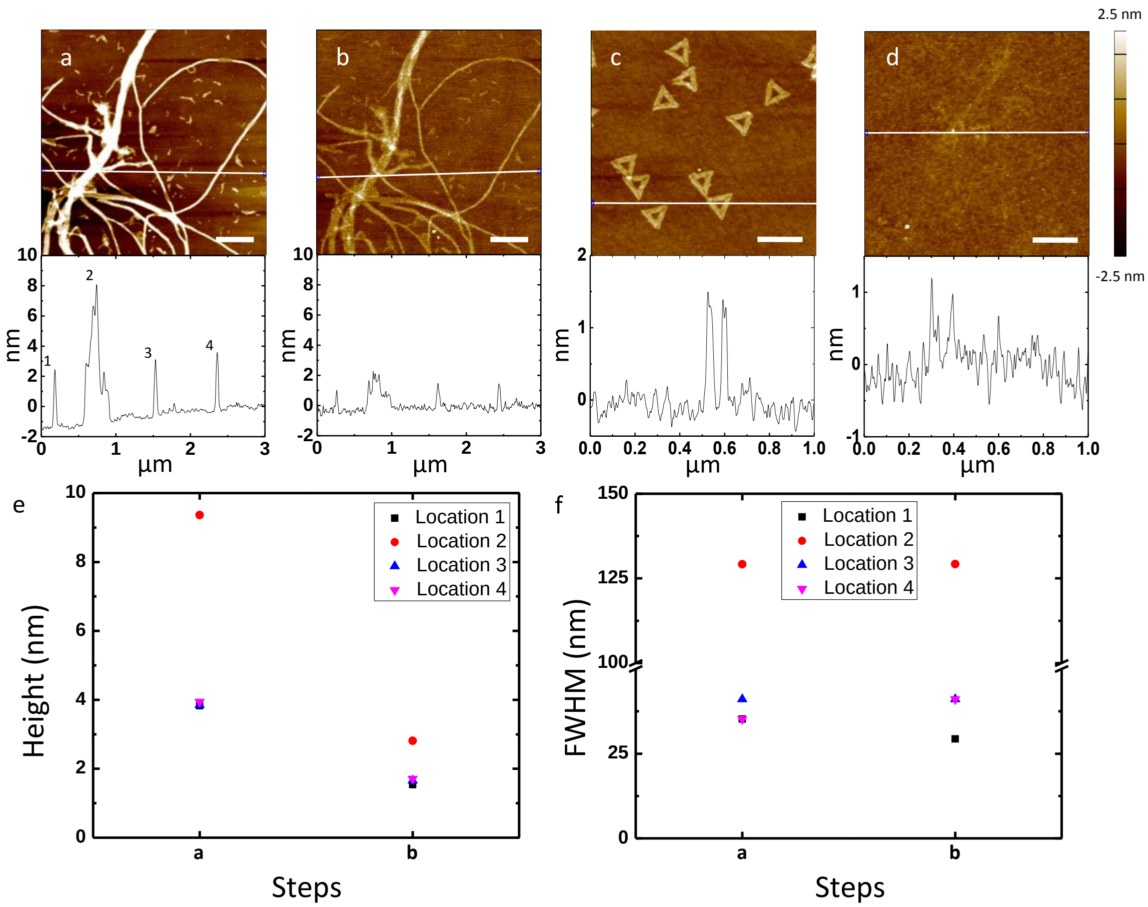


**Figure S10:** Comparison of features on DNA nanotube and origami triangle master templates with a ca. 20 nm thick $Al_2O_3$ film. AFM height images and corresponding cross-sectional analysis of DNA (a,b) nanotubes and (c,d) origami triangles deposited on silica wafers (a,c) before and (b,d) after 200 cycles of ALD of $Al_2O_3$. White lines on the AFM images indicate where the cross-sections were determined. (e) Height and (f) FWHM of the DNA nanotubes in four different locations of the AFM images from (a) to (b). Locations 1, 2, 3, and 4 correspond to 1, 2, 3, and 4 in the cross-section of the AFM image (a). Scale bars represent 500 nm (a,b) or 200 nm (c,d).

## References

(1) Rothemund, P. W. K. *Nature* **2006,** *440*, 297–302. http://www.nature.com/nature/journal/v440/n7082/suppinfo/nature04586_S1.html
